# Trypanosoma cruzi infection-induced changes in cardiac microvascular endothelial cell morphology and function

**DOI:** 10.1099/jmm.0.002095

**Published:** 2025-11-13

**Authors:** Lyndsey N. Gisclair, Douglas A. Johnston

**Affiliations:** 1 Department of Microbiology, Immunology, and Parasitology, School of Medicine, Louisiana State University Health Sciences Center, New Orleans, LA, USA; 2 Department of Medical Laboratory Science, School of Allied Health Professions, Louisiana State University Health Sciences Center, New Orleans, LA, USA

**Keywords:** cardiac, Chagas, dysfunction, endothelial, microvascular, *Trypanosoma*

## Abstract

**Introduction.** Six to seven million individuals are infected with *Trypanosoma cruzi*, the causative agent of Chagas disease. With 12,000 deaths annually, chronic Chagas disease remains a significant global health challenge due to persistent vector transmission, increasing non-vector transmission and limited therapeutic options. Chronic Chagas cardiomyopathy is a leading cause of morbidity and mortality, yet the underlying mechanisms remain poorly understood.

**Gap Statement.** Since its initial description more than 100 years ago, research efforts into the cardiomyopathy found in chronic Chagas disease have primarily focused on the contributions of immune cells, cardiomyocytes and cardiac fibroblasts, leaving a significant gap in understanding the role of microvascular endothelial dysfunction in disease progression.

**Aim.** The aim of this study was to identify any morphological or functional changes to cardiac microvascular endothelial cells induced by *T. cruzi* infection with the potential to contribute to the pathologies found in chronic Chagas disease.

**Methodology.** We cultured primary cardiac microvascular endothelial cell monolayers *in vitro* and infected them with *T. cruzi* trypomastigotes or exposed them to conditioned media collected from control or infected endothelial cells. Cells were analysed for changes in morphology and proliferation, by wound healing assays for measurements of migratory capacity and by tube-forming assay to characterize their ability to form capillary-like structures.

**Results.** We show that *T. cruzi* infection leads to the development of hypertrophic multinuclear cells, inhibits endothelial proliferation, increases endothelial migration and results in changes in several aspects of angiogenesis.

**Conclusion.** We present data to demonstrate morphological and functional changes in cardiac endothelial cells that occur as a result of *T. cruzi* infection and propose that these changes may contribute to endothelial dysfunction and the development of chronic Chagas cardiomyopathy.

## Data Summary

The authors confirm that all supporting data and protocols have been provided within the article or through supplementary data files.

## Introduction

American trypanosomiasis, also known as Chagas disease (CD), is recognized as a neglected tropical disease endemic to South America, Central America and Mexico. CD is now found worldwide, in both endemic and (increasingly) non-endemic areas, with as many as million people currently infected resulting in nearly 12,000 deaths annually [1]. CD is caused by infection with the hemoflagellate parasite *Trypanosoma cruzi*, which is primarily transmitted through the faeces of hematophagous triatomine insect vectors (Hemiptera: Reduviidae: Triatominae), aka ‘kissing bugs’. Transmission can also occur through blood transfusions, organ transplants, congenitally, laboratory accidents and contamination of food sources [2,3]. Unfortunately, in rural and underdeveloped areas where CD is most prevalent, both diagnosis of infection and access to treatment can reach levels as low as 1% [4,5]. *T. cruzi* infection manifests in two distinct phases termed acute and chronic. Treatment of acute infections is curative in the earliest stages but is increasingly less effective over time and contraindicated in the later stages of chronic disease. Additionally, treatment is limited to the use of only two currently approved drugs, benznidazole and nifurtimox, both requiring long-term use and associated with serious side effects, patient non-adherence and rapidly increasing parasite drug resistance [5,8]. Acute infection is usually self-limiting and asymptomatic or presents with mild flu-like symptoms and is thus widely undiagnosed outside endemic regions. Within weeks following an acute infection, the majority of untreated patients enter the chronic phase of infection where they are typically asymptomatic, and parasites are no longer detectable in the blood. This clinically indeterminate form can last for many years, though patients remain infected throughout their lifetime. Because *T. cruzi* can infect any nucleated cell within the body and lies dormant inside host cells for extended periods, symptomatic disease can eventually develop due to repeated, sporadic reinfections from these latent reservoirs. In endemic regions, reinfection is also common. Importantly, as many as 30% of individuals with chronic disease will eventually develop severe, life-threatening complications, often many decades later, including digestive system pathologies such as megaoesophagus and/or megacolon. However, the most common chronic manifestation by far is cardiomyopathy leading to thrombolytic events, arrhythmias and heart failure – collectively termed chronic Chagas cardiomyopathy (CCC) [9,10].

Cardiac tissue is largely composed of cardiomyocytes, fibroblasts, endothelial cells (ECs) and the mural cells required to support them. Contrary to earlier studies that identified cardiac fibroblasts as the most prevalent, recent work suggests that the most numerous cell type within cardiac tissue is actually the EC and that endothelial functions are likely more critical to cardiac function than previously thought [11,12]. The microvascular ECs that line the arterioles, capillaries and venules within the heart possess unique, tissue-specific morphological and functional characteristics [13,14]. In cardiac muscle, increased capillary density is essential to meet the dynamic demands of such a highly metabolic tissue. The microcirculation is responsible for regulating the exchange of gases, nutrients and waste products between the blood and tissues, but also controls vascular tone, immune cell recruitment and wound healing [15]. Therefore, just as a properly functioning microvasculature is absolutely essential to proper cardiac function, endothelial dysfunction is a significant driver of cardiomyopathy.

Microvascular dysfunction is implicated in a wide variety of diseases, from diabetes to Alzheimer’s disease. Cardiovascular diseases are also well represented, where EC dysfunction is associated with deleterious changes in vasomotor tone, oxidative stress responses, haemostasis, proliferation and inflammation [16]. In CCC, vascular inflammation was identified as early as 1911, with reports describing perivascular inflammation in cardiac tissue, indicative of disruption of endothelial haemostasis in the chronic phase of disease [17]. The acute response to high-level *T. cruzi* parasitaemia leads to inflammation surrounding EC, leading to thickening of the endothelial basement membrane (BM) and autoreactivity to altered BM components – both of which contribute to fibrosis and ventricular dysfunction [18,19]. Diffuse, chronic, low-level inflammatory responses over several years likely contribute to repeated, localized, ischaemic events, leading to cardiomyopathy [20,21]. Additionally, hearts from patients who died of Chagas cardiomyopathy and experimental mouse models of chronic CD revealed a diversity of pathologies associated with abnormal vasculature. Findings included regions of both increased capillary density and regions of decapillarization, lesions characterized by oedema and microaneurysm formation, regions of both vascular constriction and dilation, capillary BM thickening and increased microvascular proliferation [18,20, 22, 23]. The somewhat contradictory, simultaneous presentation of both vasoconstriction and dilation, and decapillarization and neocapillarization, suggests a complex interplay between EC functional regulation, EC responses to parasitic antigens, *T. cruzi* alteration of EC function to meet its reproductive needs and the inflammatory microenvironment generated by the persistent presence of local immune cell infiltrates [24,25]. It is therefore highly likely that the microvasculature experiences repeated, diverse and possibly conflicting signals at or near sites of infection that predispose them to dysfunction.

Angiogenesis, the generation of new blood vessels from existing vessels, is a tightly regulated, yet critical process for maintaining tissue homeostasis under both quiescent and inflammatory conditions, including the facilitation of immune cell recruitment and control of wound repair. Conversely, dysregulation of angiogenesis is implicated in a plethora of diseases, from rheumatoid arthritis to malignancies, as ECs attempt to restructure the vasculature to meet the needs of local tissues – often with unfavourable results [26]. One of the hallmarks of angiogenesis is the increased expression of the master regulator vascular endothelial growth factor (VEGF). VEGF has profound effects on multiple cell types and is a critical growth factor required for proper microvascular EC function. VEGF is also strongly implicated in numerous pathologies, making it a prime target for powerful therapeutics [27,29]. Studies have shown that *T. cruzi*-mediated cardiac inflammation results in increased expression of VEGF, and the exacerbation of angiogenesis has been linked to Chagas cardiomyopathy in both mice and humans [30,32].

Importantly, productive angiogenesis requires the careful coordination of several distinct EC activation signalling pathways resulting in changes in cellular proliferation, migration, matrix remodelling, tube formation and vessel maturation. In this report, we provide evidence that *T. cruzi* infection can induce significant angiogenesis-associated morphological and functional changes in primary cardiac microvascular ECs (cMVECs) with considerable potential to contribute to the development of the cardiomyopathy seen in chronic CD.

## Methods

### EC culture conditions

Human cMVEC (Cat. FC-0053) were purchased from Lifeline Cell Technology (Fredrick, MD, USA) and grown in EBM2 basal medium (Lonza Cat. CC-3156) supplemented with EGM-2MV SingleQuot kit (Lonza Cat. CC-4147) solution in a humidified 5% CO_2_ incubator at 37 °C. Cell culture surfaces were pre-treated with a solution of 0.5% Gelatin (Sigma-Aldrich Cat. G1393) in PBS (Gibco Cat. 10010023) to improve endothelial attachment. cMVECs were only used from passages 6–8. The human microvascular EC line HMEC-1 cells (ATCC CRL-3243) were purchased from American Type Culture Collection (Manassas, VA, USA) and maintained in MCDB131 medium (Gibco Cat. 10372019) supplemented with 10 ng ml^−1^ recombinant human epidermal growth factor (Gibco Cat. PHG0311L), 1 ug ml^−1^ hydrocortisone (Sigma Cat. H0888-1G), 10 mM glutamine (Gibco Cat. 25030081), 10% fetal bovine serum (Atlanta Biologicals Cat.) and 10 U ml^−1^ penicillin/streptomycin (Gibco Cat. 15140122) solution in a humidified 5% CO_2_ incubator at 37 °C.

### *T. cruzi* culture conditions

*T. cruzi* parasites (CL Brener strain, tdTomato-expressing [33]) were a kind gift from Dr Ben Kelly (Louisiana State University Health Sciences Center). Parasites were expanded by infecting confluent HMEC-1 monolayers in Dulbecco’s Modified Eagle Medium (DMEM) (Gibco Cat. 10313021) supplemented with 10 mM glutamine (Gibco Cat. 25030081), 10% fetal bovine serum (Atlanta Biologicals) and 10 U ml^−1^ penicillin/streptomycin (Gibco Cat. 15140122) (herein referred to as DMEM^+^). The culture media were replenished every 48 h. On day 6 of infection, *T. cruzi* tissue culture-derived trypomastigotes (TcTs) were harvested from HMEC-1 monolayers and the media were replenished. Harvesting was repeated every 48 h until the cell monolayer was no longer viable. Cell culture medium containing TcT was centrifuged at low speed (230 rcf for 5 min) to remove cell debris, and the supernatant was transferred to a new tube and centrifuged at high speed (1,500 rcf for 10 min) to pellet TcT. The cleared medium was aspirated from the cell pellet, and TcTs were resuspended in fresh DMEM. TcTs were quantified by hemocytometer and used immediately or maintained in a humidified 5% CO_2_ incubator at 37 °C with DMEM replacement every 48 h until use.

### Conditioned media collection

HMEC or cMVECs were trypsinized (Gibco Cat. 12604021) and resuspended in EGM-2MV to a cell density of 1.5×10^6^ cells ml^−1^ before seeding 1 ml of cell suspension into gelatin-coated plate tissue culture flasks. Cells were maintained in a humidified 5% CO_2_ incubator at 37 °C with media replacement every 48 h until confluent. At confluency, the media was replaced with fresh media (control CM) or media containing TcT at an m.o.i. of 15 (infected CM). After 48 h of infection, cell culture supernatants were harvested and centrifuged at high speed (1,500 rcf for 10 min) to pellet cell debris and TcT. The cleared media was transferred to new tubes and stored at −20 °C until use.

### Immunofluorescence assays

Twelve millimetre round glass coverslips (Electron Microscopy Sciences Cat. 72222–01) were placed into 12-well tissue culture plates and coated with 0.5% gelatin. cMVECs were trypsinized and resuspended in EGM-2MV to a cell density of 1×10^5^ cells ml^−1^ before seeding 1 ml of cell suspension onto coverslips. cMVEC monolayers were maintained in a humidified 5% CO_2_ incubator at 37 °C. Monolayers were mock-infected or infected with TcT at an m.o.i. of 1 or 10. At days 1 and 5 post-infection (p.i.), monolayers were fixed in 4% paraformaldehyde (Electron Microscopy Sciences Cat. 15710) for 20 min, permeabilized for 5 min with 0.1% Triton X-100 (Sigma-Aldrich Cat. T9284) and blocked with immunofluorescence blocking buffer (Cell Signaling Technology Cat. 12411) for 1 h. Coverslips were then stained with rabbit anti-VE-cadherin (VE-CAD) (Cell Signaling Technology Cat. 2500) diluted in 1:500 PBS (Sigma Cat. P3563)+0.05% Tween 20 (Sigma-Aldrich Cat. P1379) overnight at 4 °C with rocking. After overnight incubation, coverslips were stained with anti-rabbit F(ab')2 Fragment (Alexa Fluor 488 Conjugate) diluted 1:1,000 (Cell Signaling Technologies Cat. 4412) in PBS+0.05% Tween 20 for 2 h at room temperature with rocking. Coverslips were then mounted onto glass slides using ProLong Gold Antifade Mountant with DNA Stain DAPI (Invitrogen Cat. P36931). 100× and 400× magnification images were collected using an inverted Axiovert fluorescent microscope (Zeiss Group, USA). Each experiment was repeated three times (*n*=3) with 2–3 coverslips per condition.

### Cell proliferation assays

For proliferation assays with conditioned media (CM), the CellTiter 96 AQueous One Solution Cell Proliferation Assay system (Promega) was used according to the manufacturer’s instructions. HMEC or cMVECs were trypsinized (Gibco Cat. 12604021) and resuspended in EGM-2MV to a cell density of 2.0×10^5^ cells ml^−1^ before seeding 100 µl of cell suspension into 96-well gelatin-coated plates. Cell monolayers were incubated in a humidified 5% CO_2_ incubator at 37 °C overnight before being treated with 100 µl of control CM or infected CM for 48 h. After 48 h, 20 µl of CellTiter 96 AQueous One Solution reagent was added to each well and incubated for 4 h. Absorbance at 490 nm was measured on an Agilent Bio-Tek Cytation 1 Cell Imaging Multimode Reader (Santa Clara, CA, USA). Average cell counts were obtained by comparing the absorbance values of three replicate wells across three experiments (*n*=3) using a standard curve.

For *T. cruzi*-infected cell proliferation assays, cMVECs were trypsinized and resuspended in EGM-2MV to a cell density of 4×10^4^ cells ml^−1^ before seeding 500 µl of cell suspension into 48-well gelatin-coated plates. The cells were allowed to adhere to the plates overnight in a humidified 5% CO_2_ incubator at 37 °C. Monolayers were then replenished with fresh DMEM^+^ or DMEM^+^ containing TcT at an m.o.i. of 1, 5 or 10. After 24 h, cells were washed twice with DMEM^+^ to remove cell debris and unbound TcT. Monolayers were incubated for 48 h and brightfield images were collected at *T*=0 and *T*=48 h using a Cytation 1 Cell Imaging Multimode Reader. Proliferation was measured by counting the number of cells per image at *T*=0 and *T*=48 for duplicate wells across three experiments (*n*=3) using ImageJ software (NIH).

### Wound healing assays

Migration was assessed using wound healing assays employing both CM and direct infection. For CM assays, cMVECs were trypsinized and resuspended in EGM-2MV to a cell density of 2×10^5^ cells ml^−1^ before seeding 1 ml of cell suspension into 12-well gelatin-coated plates. The cells were allowed to adhere to the plates overnight in a humidified 5% CO_2_ incubator at 37 °C. After overnight incubation, monolayers were treated with control CM or infected CM for 2 h prior to wound introduction using a sterile P1000 pipette tip. After wounding, monolayers were washed twice and replenished with appropriate CM. Monolayers were maintained at 37 °C for the duration of the experiment. Brightfield images of the wounds were collected at *T*=0 and 16 h using a Cytation 1 Cell Imaging Multimode Reader. Wound boundaries were demarcated manually in ImageJ, and closure was determined by measuring the percent reduction in total wound area (in pixels) after 16 h for duplicate wells across three independent experiments (*n*=3) [34].

For *T. cruzi*-infected wound assays, cMVECs were plated as above into 12-well plates. Upon reaching confluency, monolayers were replenished with fresh DMEM^+^ or DMEM^+^ containing TcT at an m.o.i. of 1 or 10. The infection was allowed to progress for 48 h before wound introduction and closure quantification as above.

### Matrigel tube-forming assays

cMVECs were grown in T-25 culture flasks until confluency as described above. At confluency, cells were infected with TcT at an m.o.i. of 1, 5 or 10 for 24 h. After 24 h, cells were washed twice to remove unbound TcT and fresh media were added. Reduced Growth Factor Matrigel (BD Biosciences Cat. 356230) was added to a chilled 96-well plate (45 µl/well) and allowed to polymerize for 45 min at 37 °C. Infected and control cMVEC monolayers were trypsinized and resuspended in EGM-2MV to a cell density of 1×10^5^ cells ml^−1^ before seeding 100 µl of cell suspension atop each Matrigel-coated well. Cells were maintained at 37 °C for 24 h, and brightfield images of each well were collected at *T*=0, 4, 8, 12 and 24 h using a Cytation 1 Cell Imaging Multimode Reader. The mean tube length, tube numbers, branch points, loops formed and mean loop areas were determined for each condition using WimTube Tube Formation Assay Image Analysis Solution (Wimasis, 2016). All samples were assayed in duplicate across three separate experiments (*n*=3).

## Results

### Endothelial morphology

**Fig. 1.**
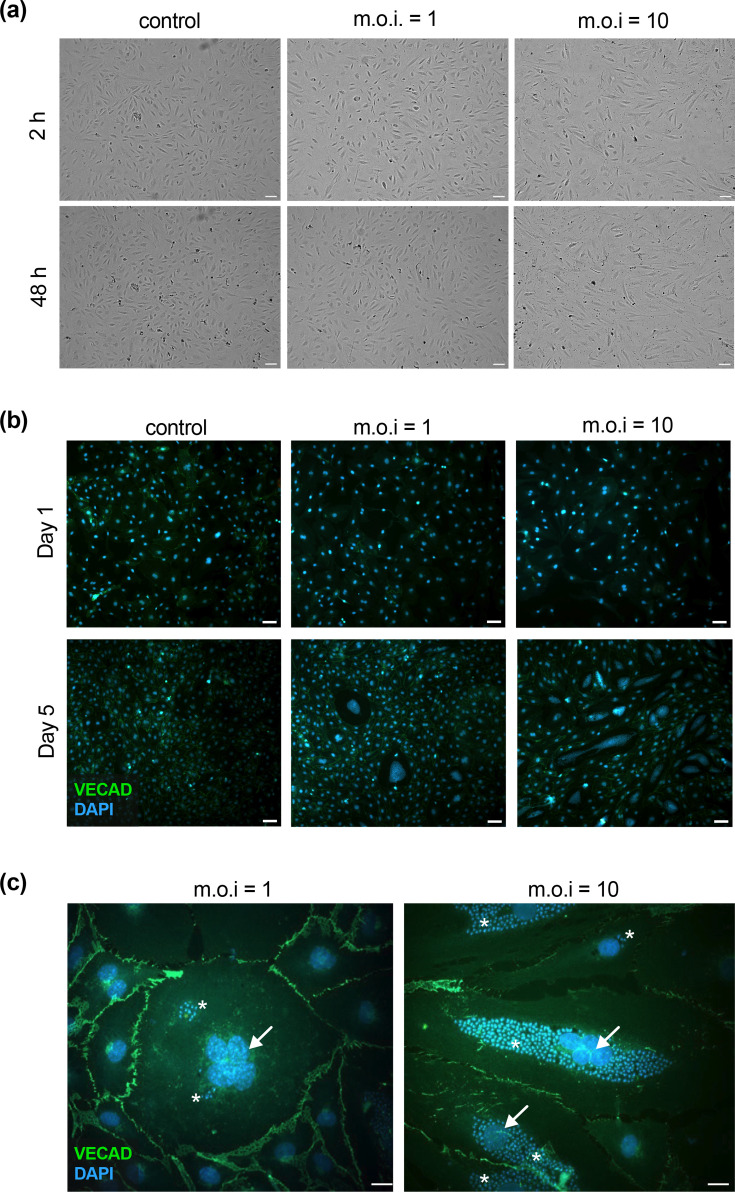
*T. cruzi* infection induces morphological changes in cMVEC. cMVEC monolayers were infected at m.o.i. of 1 and 10. (**a**) As early as 2 h p.i., cells begin to show signs of activation, including cell spreading and elongation. 100×, scale bars=100 µm. (**b**) Monolayers were fixed at days 1 and 5 p.i. and stained for VE-CAD (Alexa488, green) and nuclei (DAPI, blue). 100× magnification showing the presence of giant cMVEC. Scale bars=100 µm. (**c**) 400× magnification of giant cMVEC at 5 days p.i. with arrows indicating multiple nuclei, asterisks (*) indicate parasite amastigote nests. Scale bars=20 µm.

*T. cruzi* readily infects ECs, but there are no data available on the effects of infection in human cardiac ECs. Therefore, we first evaluated changes in EC morphology during *T. cruzi* infection. Monolayers of cMVEC were mock-infected or infected with *T. cruzi* trypomastigotes at an m.o.i. of 1 or 10 to mimic both low-grade infections often observed during CCC and heavier infections to more closely reflect parasite load during acute stages. At days 1 and 5 p.i., cells were fixed and prepared for immunofluorescent microscopy. VEGF was originally described as a vascular endothelial permeability factor secreted by tumour cells [35,37]. Subsequent studies showed that VEGF signalling induces the phosphorylation and endocytosis of vascular endothelial cadherin (VE-CAD), the primary structural component of endothelial adherens junctions, resulting in the rapid dissolution of EC-cell contacts [38,39]. Therefore, we stained infected monolayers to look for changes in VE-CAD expression and localization. Though we identified minor disruptions in some infected cell contacts at low m.o.i., we could not find any significant changes, and the vast majority of cell–cell contacts appeared to be intact, similar to controls (Fig. 1). Notably, however, infected cells showed rapid overall morphological changes within hours of infection, including cell spreading and elongation (Fig. 1a). At 5 days p.i., we observed numerous hypertrophic, multinucleated cells throughout infected monolayers, with the frequency increasing with increasing m.o.i. (Fig. 1b). For these experiments (*n*=3), we counted between 1,500 and 2,000 total cells per condition and found multinucleated cells at absolute frequencies of 0.09% at m.o.i. 1 and 0.25% at m.o.i. 10. However, when normalized to infection rate, ~3% of infected cells were multinucleated at m.o.i. 1 (average infection rate 3%), compared to only 0.6% of infected cells at m.o.i. 10 (average infection rate 40%). Thus, although the absolute frequency of multinucleated cells increased with higher infection burden, the proportion of infected cells that underwent multinucleation was markedly reduced at higher m.o.i. Hypertrophic, multinucleated cells were not observed in either neighbouring uninfected cells or in any cell under control conditions. This suggests that the development of multinuclear cells is dependent on infection rate, perhaps due to either the proximity of infected cells to each other or the number of invading parasites found within a given cell. It remains to be determined if these changes occur as a result of either cell–cell fusion events or by blocking host cell cytokinesis. Interestingly, at high m.o.i., the amastigotes often appear to form a single large perinuclear mass distinct from the surrounding cytoplasm, while at low m.o.i., individual nests appear randomly throughout the host cell cytoplasm (Fig. 1c).

### Endothelial proliferation

**Fig. 2.**
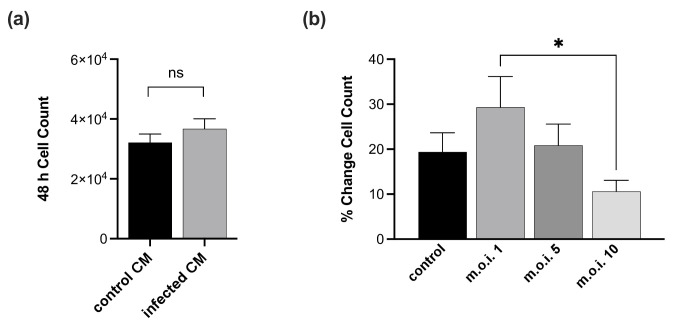
*T. cruzi* infection inhibits cMVEC proliferation. cMVECs were seeded at low density to prevent contact inhibition. (**a**) Monolayers were treated with CM collected from control or infected cMVEC monolayers. Results are reported as cell counts 48 h post-treatment. (**b**) cMVEC monolayers were mock-infected or infected with TcT at an m.o.i. of 1, 5 or 10. The number of cells was counted and the results are reported as the per cent change in cell count based on *t*=0 for each sample. Vertical bars represent mean±sem with significant differences (*P<0.05, **P<0.01, ***P<0.001, ****P<0.0001) based on one-way ANOVA followed by Šídák’s multiple comparisons test. Both experiments were repeated three times.

As endothelial proliferation is required for functional angiogenesis, we evaluated the effect of *T. cruzi* infection on EC proliferation. Due to the importance of autocrine and paracrine signalling in the maintenance of vascular homeostasis, we tested the effects of CM collected from both cMVEC control and *T. cruzi*-infected samples. No significant differences in cMVEC proliferation were observed after 48 h of treatment with CM (Fig. 2a). We then assessed cMVEC proliferation during direct infection with *T. cruzi*. Monolayers of cMVEC were infected at an m.o.i. of 0, 1, 5 or 10. Images were collected from the same location within each well over a 48-h period, and the cells in each image were counted. Because the infection protocol takes 2 h and the cell morphologies change significantly, affecting the number of cells per field, it is difficult to compare control versus infected cells directly. However, when we measured the percent increase in cell numbers among individual samples, we found a significant dose-dependent reduction in cell counts in infected cMVEC (Fig. 2b), indicating that *T. cruzi* impacts the ability of infected cells to proliferate. These results are not surprising and support our findings of hypertrophic infected cells, which are likely unable to proliferate due to heavy parasite burden.

### Endothelial migration

**Fig. 3.**
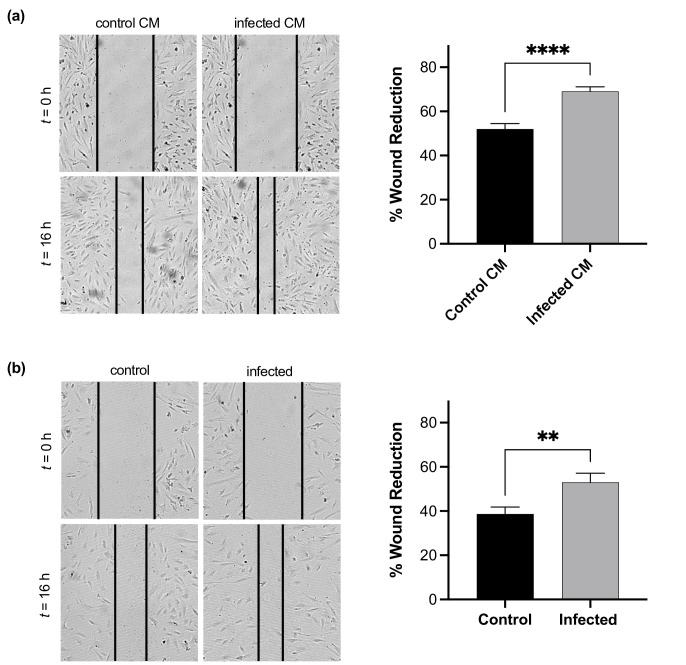
*T. cruzi* infection induces increased EC migration. Confluent cMVEC monolayers were wounded and treated with CM collected from infected and control cMVEC (**a**) or were infected at an m.o.i. of 1 for 48 h prior to wound introduction. (**b**) Representative bright field images were collected at 0 and 16 h post-wound introduction at 400× magnification and total wound areas were measured. Results reported as per cent decrease in total wound area after 16 h. Vertical bars represent mean±sem with significant differences shown with asterisks (**P<0.01, ****P<0.0001) based on Student’s t-test. Each assay was repeated three times.

We utilized a wound healing assay to determine if *T. cruzi* infection altered endothelial migration, another important component of angiogenesis as cells must undergo directed migration to form new vessels. Preliminary studies were performed to identify an optimum timepoint for measuring migration in cMVEC. We found that timepoints between 12 and 18 h post-wounding revealed significant levels of migration without the risk of complete wound closure. This also limits the contribution of cell proliferation to the results, as the average doubling time for cells used in this study was between 28 and 36 h. We first explored the effect of cell–cell signalling on cMVEC migration using CM from untreated and infected EC. For scratch wound assays, confluent cMVEC monolayers were wounded and then treated with control or infected CM for 16 h. We found a significant increase in wound reduction in cMVEC treated with infected CM, suggesting infection results in the release of soluble mediators that lead to enhanced activation of EC migration (Fig. 3a). We then directly infected cMVEC monolayers at an m.o.i. of 1 for 48 h prior to wounding. At 48 h p.i., *T. cruzi* trypomastigotes that have successfully invaded a host cell will have differentiated into amastigotes and will undergo replication for the next 2–3 days. At 16 h post-wound generation, we saw a significant increase in wound area reduction (Fig. 3b). This suggests that infected ECs experiencing active amastigote replication within their cytoplasm also gain an enhanced migratory phenotype.

We then expanded our migration assay to include a time-course and to determine if infectious dose size had any effect. cMVEC monolayers were mock-infected or infected at an m.o.i. of 1 or 10 as described above. For this experiment, we utilized the IncuCyte Platform and Software, which utilizes an advanced protocol with robotic wound creation and continuous monitoring over time to measure relative wound density (RWD), which is a measure of the spatial cell density within the wound area relative to the spatial cell density outside of the wound area (ranging from 0 to 100%). It is also designed to be self-normalizing for changes as a result of cell proliferation [40]. We measured the RWD every 3 h over a 24-h period and preliminary results (*n*=1) showed that in basal media, control cells reached closure densities of 47%, while infected cells reached 53–55% (Fig. S1A). Interestingly, we found that when the media was supplemented with growth factors (EGM2-MV), the wound densities for control cells increased to only 52%, while infected cells at both m.o.i. reached over 76% (Fig. S1B, available in the online Supplementary Material). This finding suggests that infected cells are hyperresponsive to growth factors as compared to controls. Most surprisingly, we observed both uninfected and infected ECs migrating into the open wound area. This clearly demonstrates that infected cells retain their migratory capacity during amastigote replication and that these increases in migration are not simply due to a paracrine signalling phenomenon activating neighbouring cells (Fig. S2).

### Endothelial tube formation

**Fig. 4.**
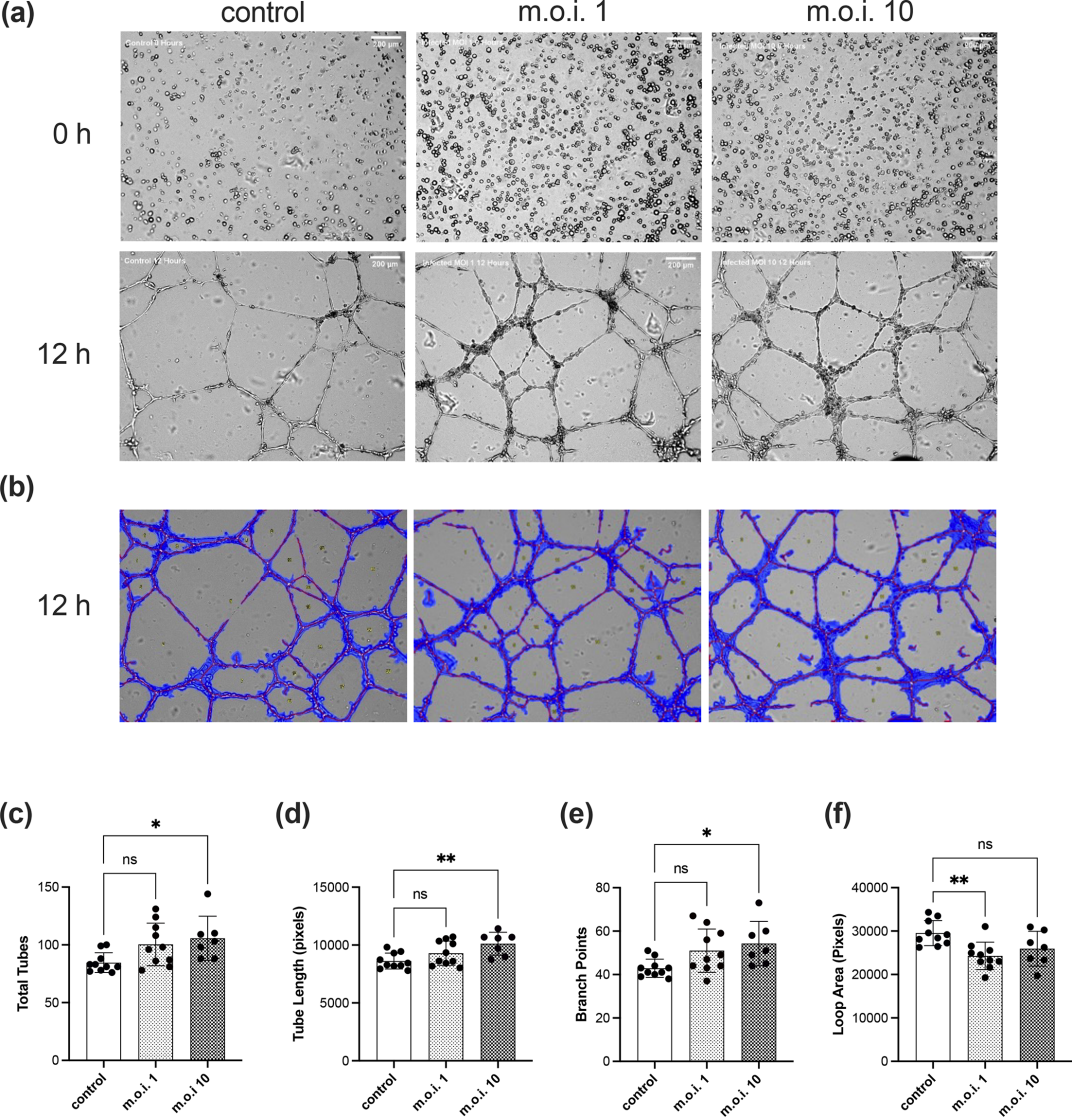
*T. cruzi* infection affects tube formation. cMVECs were mock-infected or infected with TcT at an m.o.i. of 1 or 10. Cells were seeded onto solidified Matrigels and monitored for their ability to form tube structures. (**a**) Bright-field images were captured using 400× magnification at 12 h post-seeding and analysed for tube formation. (**b**) Representative images and tube formation analysis parameters. Covered image area (blue), tubes (red), branch points and loops. (**c**) Total number of tubes per image. (**d**) Quantification of tube length per image in pixels. (**e**) Number of branch points per image. (**f**) Quantification of loop area per image in pixels. Vertical bars represent mean±sd and significant differences shown with asterisks (*P<0.05, **P<0.01, ***P<0.001, ****P<0.0001) based on one-way ANOVA. All experiments were repeated three times.

Tube formation and the subsequent maturation into viable vessels is the ultimate goal of angiogenesis. Using a Matrigel assay, we evaluated the ability of infected cMVEC to form tube structures during the amastigote replication stage of infection. cMVEC monolayers were infected at an m.o.i. of 1 or 10 for 48 h prior to seeding onto Matrigel, and tube formation was monitored over a 24-h period. Infected cMVEC formed an organized interconnected network of tubes by 8–12 h after seeding, qualitatively similar to control cells (Fig. 4a). To identify structural changes in tube formation during infection, images were analysed using WimTube software (Fig. 4b). We found that cMVEC infected at the higher m.o.i. showed significant increases in the total number of tubes formed, total overall tube length and the number of branch points (Fig. 4c–e). We found no significant differences in the total number of loops formed between any conditions. Additionally, while there was a trend toward increases in all aspects under both infected conditions, the changes for cells infected at the lower m.o.i. were not statistically significant at this time point, with the one exception of smaller average loop area (Fig. 4f). Taken together, these data suggest that while low-level *T. cruzi* infection results in minor alterations to newly formed tubular EC networks, the increased stress of higher parasite burden has significant effects on multiple aspects of endothelial function and the angiogenic process.

## Discussion

It is well-established that ECs from different tissues have unique characteristics, and their responses to external stimuli vary widely, and thus careful consideration should be given to cell origins when implementing EC into biomedical research protocols (reviewed in [14,41]). Despite this, the vast majority of studies of vascular involvement in *T. cruzi* infection have employed either human umbilical vein ECs, murine cells or other less-appropriate cell lines [24,42,46]. We have chosen to use primary human cardiac ECs in this investigation as we believe they offer a more physiologically relevant model for studying this disease. Our preliminary analysis of the secretome of a *T. cruzi*-infected 3D cardiac organoid culture (comprising primary human cardiac myocytes, fibroblasts and MVEC) revealed increases in multiple inflammatory cytokines, including a 3.5-fold increase in VEGF expression (data not shown). Subsequent STRING database mapping of upregulated proteins suggested strong associations with CD, chronic inflammatory processes, endothelial function and cellular migration. Not surprisingly, VEGF was found to be the most interconnected between all up-regulated proteins identified. This led us to look further into potential downstream effects of VEGF signalling, including cellular morphology and angiogenic functions.

The observation that *T. cruzi* infection can lead to the development of enlarged, multinucleate EC was unexpected. The formation of multinucleate cells is commonly the result of cell–cell fusion or blocks in cell cycle events such as cytokinesis. And while pathogen-induced host cell syncytia formation due to cell–cell fusion is well documented in viral infections [47], there are relatively few reports of multinuclear host cells associated with parasitic infections. Infection with *Leishmania* spp., the aetiologic agents of both cutaneous and visceral leishmaniasis, induces the formation of multinucleate giant cells both *in vitro* and *in vivo* – the result of monocyte/macrophage cell–cell fusion [48,50]. It has been shown that *T. cruzi* trypomastigotes can stretch the plasma membrane of host cells both during invasion and when exiting the cell, causing damage that could potentially lead to membrane fusion events, but no definitive evidence of this has been reported [51]. More likely, *T. cruzi* infection leads to a block in host cell cytokinesis, as has recently been shown to occur in infected human foreskin fibroblasts [52]. Similarly, *Toxoplasma gondii* and *Plasmodium falciparum* block host cell cytokinesis, leading to multinucleate cell formation in bovine umbilical vein ECs and HepG2 carcinoma cells, respectively [53,54]. Another parasite, the helminth *Trichinella spiralis*, induces the formation of nurse cells in striated muscle. Nurse cells are multinucleate and express high levels of VEGF, allowing for increased pathogen survival and replication [55]. Importantly, because many of these findings were based solely on *in vitro* infections and diverse cell types, further studies are warranted to determine the mechanism and, ultimately, relevance of this phenomenon in CD.

Increased fibrosis and hypertrophy are hallmarks of CCC, and ECs have been shown to contribute to fibrotic disease in other organs, both directly and indirectly [56,57]. However, while thickening of EC BMs has been demonstrated in Chagas fibrosis by others, we found no evidence of increased gene expression for several BM proteins in *T. cruzi-*infected MVEC, including several collagens, fibronectin and vimentin in our study (data not shown). This supports the widely held view that cardiomyocytes and fibroblasts, not EC, are responsible for the bulk of fibrotic changes and tissue remodelling in cardiac tissues [58,60]. We also tested the ability of *T. cruzi* to induce endothelial-to-mesenchymal transition (EndMT) in MVEC, which results in the differentiation of EC into a highly fibrotic phenotype. However, while cMVECs treated with TGF*β* and IL-1*β* showed increased expression of the mesenchymal markers *α*-SMA and COL1A1, as well as the loss of endothelial markers VE-CAD and CD31, *T. cruzi*-infected cells did not (data not shown). These results show that direct infection of cMVEC alone does not likely contribute to increased deposition of extracellular matrix (ECM) components. This also suggests that infection alone is not sufficient to drive EndMT in these cells but does not eliminate the possibility that additional cell–cell interactions, with perhaps cardiomyocytes, fibroblasts or mural cells, may drive subsequent EC contributions to fibrosis.

VEGF is sequestered in the ECM and increases in fibronectin and fibrinogen can lead to VEGF-dependent EC proliferation and migration [60,63]. We asked if *T. cruzi* infection affects proliferation rates in EC and found, as suspected, that direct infection has a deleterious effect on EC proliferation. We also found no significant difference between cells exposed to CM from control or infected cells. Other studies have reported contrasting findings within this context. Hassan *et al*. showed that *T. cruzi* infection induced proliferation in cardiac vascular smooth muscle cells in an endothelin (ET-1) dependent manner [64]. ET-1 is a highly vasoactive endothelial-specific peptide strongly associated with inflammation, cardiac remodelling and myonecrosis. But while some studies have suggested a protective effect of ET-1 in early stages of *T. cruzi* infection [65,66], the current study does not address this aspect.

While we found that exposure to CM collected from infected EC resulted in increased migration rates, our finding that direct *T. cruzi* infection causes increased migration in wounded EC monolayers as compared to uninfected monolayers is quite surprising. We plan to explore these novel results further to uncover any impacts infected EC may have on their uninfected neighbours. And though here we have only measured overall migration rates, we have not determined specific differences between infected and uninfected cells. We also found that migration rates were further increased in supplemented media but have yet to determine if this is simply a result of overall EC activation or dependent upon the activities of a specific growth factor (e.g. VEGF). Morris *et al*. showed that *T. cruzi* infection of EC results in changes to the underlying ECM that can have paracrine effects on neighbouring uninfected cells, particularly effects on cellular morphology [44]. This may help to explain changes in cell motility, as changes to the underlying matrix can have profound effects on integrin and other adhesion molecule expression required for cell motility. Somewhat in contrast, Silveira *et al*. found that *T. cruzi* infection blocked migration and increased proliferation in a breast cancer cell line [67]. This also confirms that results are often cell-type specific, and this should be taken into consideration when interpreting experimental data.

As the regulation of angiogenesis is vital for tissue homeostasis and repair, especially in an organ as highly vascularized as the heart, we employed a Matrigel assay to evaluate the effects of *T. cruzi* infection on cMVEC tube-forming function. Successful endothelial tube formation requires multiple cellular processes, including BM reorganization, cell detachment and migration and cell–cell adhesion, and is regulated via multiple signalling pathways (reviewed in [68]). During this process, ECs form branched vessels that ultimately mature into functional capillaries. Excessive, disorganized or immature vessels fail to function properly and can lead to pathology. Our study found that *T. cruzi*-infected cMVECs are capable of forming these structures *in vitro*, but with several distinct differences that may contribute to impaired function in chronic Chagas. That infected EC may participate in the formation of new vessels within damaged tissues may provide additional reservoirs for increased inflammation in areas of wound repair. It is also critically important to consider that endothelial dysfunction is strongly tied to age-related vascular diseases associated with impaired angiogenesis and senescence, and this is likely to have great implications in the development of CCC [69,70].

A recent analysis of Chagas patients with heart disease, but without heart failure, found that the peripheral cardiac vasculature showed no signs of dysfunction as compared to those who did experience heart failure [71]. Also, Teixeira *et al*. showed that *T. cruzi* infection resulted in anti-angiogenic effects [72]. These studies offer further support to the idea that Chagas cardiomyopathy is complex, variable and dynamic, and that host immunity, environment and parasite strain are just a few of the multiple factors involved in this disease. Of course, *in vivo*, there is a strong immune component to chronic Chagas involving repeated bouts of inflammation. Additionally, several studies suggest that this leads to autoreactivity (reviewed in [73]). Autoreactivity in the development of vascular lesions in CCC is not a new idea. Originally described as ‘allergic phenomena’ in a review of several studies from the mid-20^th^ century [17], it is widely accepted that parasite-induced host reactivity plays a role in the development of endothelial dysfunction. This study looks strictly at parasite–endothelial interactions and does not incorporate other cell types, including immune cells, so further investigation is certainly supported.

Currently, treatments for Chagas disease are insufficient to meet the needs of those suffering from chronic disease. Only a handful of new potential therapies are currently undergoing clinical trials [74]. One of these drugs, AN15368, showed very promising early results in a non-human primate model of long-term infection [75]. Another, fexinidazole, used to treat *T. brucei* infections, has also shown some efficacy, but there are issues with tolerance and long-term parasite clearance [76]. Repositioning of older, already approved drugs and combination therapies is likely the most time- and cost-effective path to ameliorating this disease. Indeed, several studies are looking at the effectiveness of combination therapies with antifungals [77,78]. Intriguingly, Nisimura *et al*. recently showed that VEGF inhibition increases the efficacy of benznidazole treatment in mice [79]. And from our viewpoint, as the endothelium is so tightly bound to cardiac homeostasis, more studies are needed that consider the contributions of the microvasculature to Chagas cardiomyopathy. This study, along with future investigations, aims to provide further insight into this aspect of a deadly and neglected disease, in hopes of identifying new potential targets for prevention and treatment.

## Supplementary material

10.1099/jmm.0.002095 Uncited Supplementary Material 1.

## Abbreviations

BM: basement membrane
CCC: chronic Chagas cardiomyopathy
CD: Chagas disease
CM: conditioned media
cMVEC: cardiac microvascular endothelial cell
DMEM: Dulbecco’s Modified Eagle Medium
EC: endothelial cell
ECM: extracellular matrix
p.i.: post-infection
RWD: relative wound density
TcT: tissue culture-derived trypomastigote
VEGF: vascular endothelial growth factor
