# Supplementary material for: Trypanosoma cruzi infection-induced changes in cardiac microvascular endothelial cell morphology and function
Source: J Med Microbiol. 2025 Nov 13;74(11):002095. doi: 10.1099/jmm.0.002095 (PMC12614368; doi:10.1099/jmm.0.002095)
Supplement: Uncited Supplementary Material 1. [file jmm-74-02095-s001.pdf]

Supplemental Data

Figure S1-S2. Kinetic Wound Healing Assays

cMVEC were grown in gelatin coated T-25 tissue culture flasks with media replacement every 48 hours until reaching confluency. At confluency, cells were infected with TcT at an MOI of 1, 5 or 10 overnight, then cells were washed to remove excess TcT. Infected and control cMVEC were then trypsinized and resuspended at 3x10^5 cells/ml in DMEM before seeding 100 µl of each condition into gelatinized 96 well Incucyte ImageLock plates (Essen Bioscience Cat. #4379). At 48 hours post infection, monolayers were wounded using the Incucyte 96 well Woundmaker Tool (Sartorius Cat. 4563) and cell debris washed away before replacing with 100 µl of basal DMEM or growth EGM-2MV. Cells were maintained in a humidified 5% CO_2 incubator at 37°C for the duration of the experiment. Two images of the wound were collected per well every three hours using a Sartorius IncuCyte SX5 Live Imaging System (Göttingen, Germany). Images were analyzed by the IncuCyte Live-Cell Imaging and Analysis Software to acquire data on the relative wound density (wound area occupied by cells) at each image collection for four to six replicate wells across a single experiment (n=1).

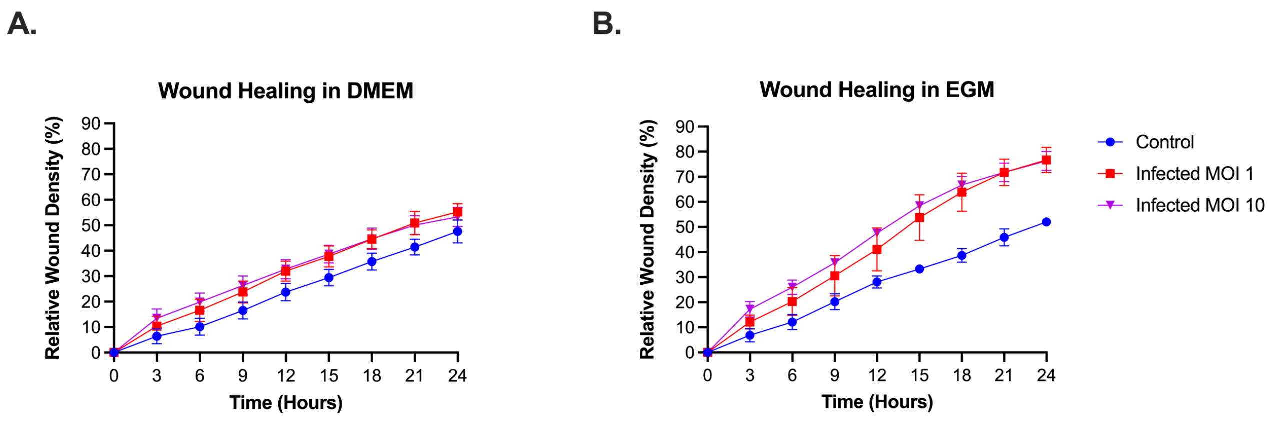

Supplemental Figure S1. Infected cMVEC Initiate Earlier Wound Healing. cMVEC were mock-infected or infected at an MOI of 1 or 10 for 48 hours prior to wound introduction. Monolayers were maintained in basal media and Brightfield and Orange channel images were collected every 3 hours. Total wound area was measured at each time point. A-B. Wound closure kinetics over 24 hours. Results are reported as percent relative wound density. Vertical bars represent Mean +/- SD.

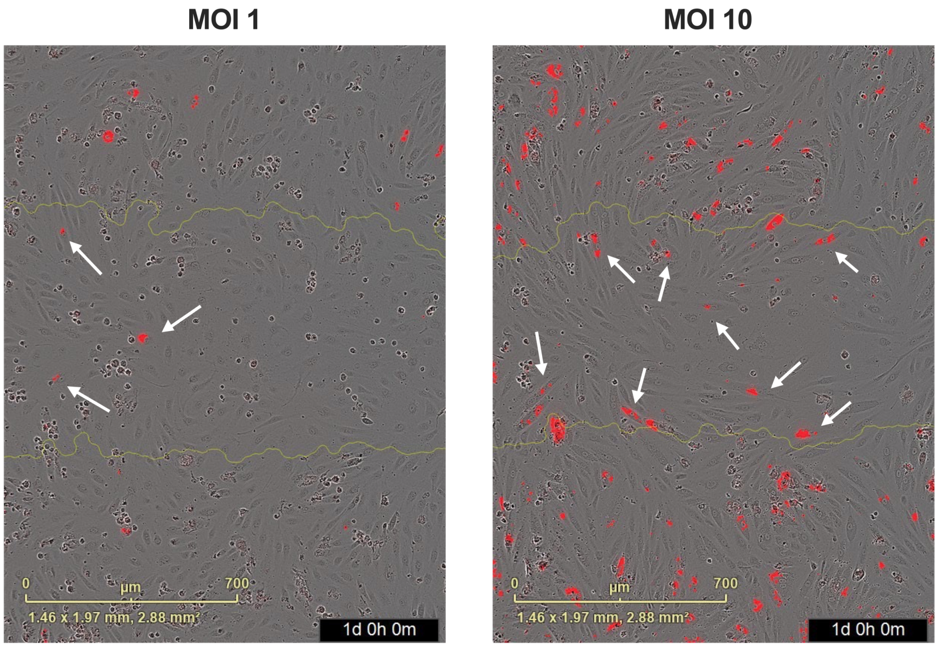

Supplemental Figure S2. Infected cMVEC Migrate into Wounded Area. Representative merged bright field and orange channel images from kinetic wound healing assay. cMVEC monolayers were infected at an MOI of 1 or 10 for 48 hours prior to wound introduction and maintained in basal media for the duration of the experiment. Yellow outline indicates the initial wounded area at T=0. White arrows indicate infected cMVEC with TdTomato-expressing *T. cruzi* amastigote nests that have migrated into the wounded area by 24 hours.
